# Methanol Extract of* Holarrhena antidysenterica* Inhibits the Growth of Human Oral Squamous Cell Carcinoma Cells and Osteoclastogenesis of Bone Marrow Macrophages

**DOI:** 10.1155/2017/7272947

**Published:** 2017-11-22

**Authors:** Heein Yoon, Junhee Park, Kwang-Kyun Park, Jin Kim, N. Champika Bandara, B. M. R. Bandara, Wanninayake M. Tilakaratne, Won-Yoon Chung

**Affiliations:** ^1^Department of Applied Life Science, The Graduate School, Yonsei University, 50-1 Yonsei-ro, Seodaemun-gu, Seoul 120-749, Republic of Korea; ^2^Department of Oral Biology, Oral Cancer Research Institute, BK21 PLUS Project, Yonsei University College of Dentistry, 50-1 Yonsei-ro, Seodaemun-gu, Seoul 120-749, Republic of Korea; ^3^Department of Oral Pathology, Yonsei University College of Dentistry, 50-1 Yonsei-ro, Seodaemun-gu, Seoul 120-749, Republic of Korea; ^4^Postgraduate Institute of Science, University of Peradeniya, 20400 Peradeniya, Sri Lanka; ^5^Department of Chemistry, Faculty of Science, University of Peradeniya, 20400 Peradeniya, Sri Lanka; ^6^Department of Oral Pathology, Faculty of Dental Sciences, University of Peradeniya, 20400 Peradeniya, Sri Lanka

## Abstract

Oral squamous cell carcinoma (OSCC) frequently invades mandibular bone, and outcomes for treatment with surgical resection are typically poor, ultimately resulting in death.* Holarrhena antidysenterica* L. (Apocynaceae), distributed throughout Sri Lanka and India, has been used as a folk remedy to treat various diseases. Treatment with methanol extract of* H. antidysenterica* bark (HABE) inhibited cell viability and BrdU incorporation and induced apoptotic cell death in Ca9-22 gingival and HSC-3 tongue SCC cells. Flow cytometric analysis indicated that HABE treatment preferentially induces apoptotic cell death via increasing the sub-G1 peak in Ca9-22 cells and cell cycle arrest at the G1 phase in HSC-3 cells. HABE treatment in the presence of zVAD-fmk, a pan-caspase inhibitor, rescued cell viabilities in both OSCC cell lines. The ratio of Bax to Bcl-2 increased with reductions in the Bcl-2 protein expression, and the activation of caspase 3 and subsequent cleavage of PARP was detected in HABE-treated Ca9-22 and HSC-3 cells. Furthermore, HABE treatment at noncytotoxic concentrations inhibited osteoclast formation in RANKL-stimulated bone marrow macrophages. Taken together, HABE possesses the inhibitory activity on the growth of OSCC cells and antiosteoclastogenic activity. Therefore, HABE may be a promising alternative and complementary agent for preventing and treating OSCC.

## 1. Introduction

Oral squamous cell carcinoma (OSCC) is the most frequently diagnosed malignant cancer in the head and neck region, accounting for approximately 95% of oral cancers [1]. The annual incidence of OSCC ranges from 400,000 to 500,000 new cases worldwide and has varied from less than 2 per 100,000 in the Middle East to 10 per 100,000 in USA and over 20 per 100,000 people in India [2, 3]. Southeast Asia, including India, Pakistan, Bangladesh, and Sri Lanka, has a high prevalence of OSCC due to cultural practices, such as betel-quid chewing and distinct patterns of tobacco and alcohol use. Thus, OSCC is a public health problem with a high treatment cost as well as a high mortality [4]. Tongue carcinoma is the leading site for OSCC in India, and the buccal mucosa and gingiva are the next most common sites in Southeast Asia [5]. In particular, gingival squamous cell carcinomas frequently invade mandibular bone and should be treated by surgical resection [6]. However, the treatment results are typically poor; nearly 70% of cases recur at the primary lesion site and ultimately result in death [7]. Bone-targeting agents, such as nitrogen-containing bisphosphonates, are also applied to OSCC patients to block cancer-related bone destruction. However, long-term administration of aminobisphosphonates causes severe osteonecrosis of the jaw, characterized by exposure of mandibular or maxillary bone [8]. Therefore, novel agents with anticancer and antibone resorptive activities are required for improving survival rate and quality of life in OSCC patients.

Phytochemicals and extracts derived from medicinal plants have been noted as promising cancer-preventive agents against several cancers because of their low toxicity and the accumulating data supporting their beneficial health effects [9].* Holarrhena antidysenterica* L. (Apocynaceae), which is distributed throughout Sri Lanka and India up to an altitude of 3,500 ft, has been used as a folk remedy for treating various diseases, including diarrhea, stomach pain, and dysentery, in India [10]. Seed extract of* H. antidysenterica* has antidiabetic, antihyperglycemic, and antihyperlipidemic activities [11]. The methanol extract of* H. antidysenterica* leaves, which has a high level of total phenolic content, scavenges reactive oxygen species (ROS) that otherwise participate in every step of carcinogenesis by causing intrinsic oxidative stress [12, 13]. The ethanol extract of* H. antidysenterica* leaves has cytotoxic activity against OVCAR-5 (ovary), HT-29 (colon), SK-N-MC (neuroblastoma), HEP-2 (liver), COLO-205 (colon), NIH-OVCAR-3 (ovary), and A-549 (lung) cancer cell lines [14]. However, the effect of* H. antidysenterica *bark extract on OSCC cell growth and bone invasion has not previously been studied.

In this study, we evaluated the preventive and therapeutic potential of* H. antidysenterica* stem and bark extract (HABE) by investigating its antiproliferative and apoptosis-inducing activity in Ca9-22 gingival SCC and HSC-3 tongue SCC cells as well as its antiosteoclastogenic activity in bone marrow macrophages, which act as osteoclast precursors.

## 2. Materials and Methods

### 2.1. Materials

Fetal bovine serum (FBS), Dulbecco's modified Eagle's Medium (DMEM), 0.25% trypsin-EDTA, and antibiotic-antimycotic mixture were purchased from Gibco BRL (Rockville, MD, USA). Phosphate-buffered saline (PBS) was purchased from Bio-Solution (Suwon, Korea). 3-(4,5-Dimethylthiazol-2-yl)-2,5-diphenyltetrazolium bromide (MTT) and dimethyl sulfoxide (DMSO) were purchased from Sigma-Aldrich (St. Louis, MO, USA). zVAD-fmk was purchased from Enzo Life Sciences (Farmingdale, NY, USA). Bicinchoninic acid (BCA) protein assay reagent and enhanced chemiluminescence (ECL) kits were obtained from Pierce (Rockford, IL, USA) and Amersham (GE Healthcare, UK), respectively. Anti-human Bcl-2, procaspase 3, and PARP antibodies were obtained from Cell Signaling Technology (Beverly, MA, USA). Anti-human Bax and GAPDH antibodies and horseradish peroxidase-conjugated anti-rabbit and anti-mouse second antibodies were purchased from Santa Cruz Biotechnology (Santa Cruz, CA, USA). All reagents used in this study were analytical grade.

### 2.2. Preparation of Methanol Extract of* H. antidysenterica* Bark (HABE)

Dried bark of* H. antidysenterica* was purchased from an herbal drug store at Kurunegala in the North-Western Province of Sri Lanka. The commercial plant sample was authenticated by a chemotaxonomic procedure and by comparison with an authentic voucher specimen (# 4539) found in the National Herbarium, Peradeniya, Sri Lanka. The dried bark was pulverized and the powder (60 g) was extracted with methanol (400 ml) in a Soxhlet apparatus for 12 h after defatting with hexane (400 ml, 12 h, Soxhlet). The extract was filtered under vacuum through a Whatman No. 1 filter paper and evaporated to dryness under reduced pressure using a Rotavapor (Heidolph Laborta 4000). HABE (2.0 g, 3.3%) was stored at −20°C. HABE for next experiments was dissolved with DMSO and diluted with serum-free media.

### 2.3. Cell Culture

Ca9-22 gingival and HSC-3 tongue SCC cells were cultured in DMEM supplemented with 10% FBS and 1% antibiotic-antimycotic mixture at 37°C in a humidified atmosphere with 5% CO_2_. Murine bone marrow macrophages (BMMs) were isolated from the tibiae of 4-week-old ICR mice as previously described [15], and they were cultured with *α*-MEM containing M-CSF (30 ng/ml), 10% FBS, and 1% antibiotic-antimycotic mixture in a humidified atmosphere with 5% CO_2_ at 37°C.

### 2.4. Cell Viability and Proliferation Assay

Ca9-22 and HSC-3 cells (1 × 10^3^ cells/well) were seeded in 96-well plates and cultured in DMEM containing 10% FBS for 24 h. The attached cells were incubated with the indicated concentrations of HABE in serum-free media for 24 and 72 h. Control cells were exposed to media with 0.1% DMSO. Cell viability was assessed using an MTT assay. The cells were further incubated at 37°C for 4 h with MTT solution (0.5 mg/ml, 20 *μ*l/well). DMSO (200 *μ*l/well) was added to each well to dissolve formazan. The absorbance was measured at 570 nm using a microplate reader (Bio-Rad Lab, Hercules, CA, USA). The amount of the newly synthesized DNA was measured using a 5-bromo-2′-deoxyuridine (BrdU) cell proliferation assay kit according to the manufacturer's protocol (Roche Applied Science, Penzberg, Germany).

### 2.5. Cell Cycle Distribution Analysis

Ca9-22 or HSC-3 cells (5 × 10^5^ cells) were seeded in six-well culture plates and incubated overnight. The cells were treated with different concentrations of HABE in serum-free media for 24 h. The cells were harvested with trypsin-EDTA, washed with PBS, and fixed with ice-cold 70% ethanol overnight at 4°C. The cells were washed with PBS again and incubated in propidium iodide solution containing 1 mg/ml RNase A, 0.1% Triton-X, and 0.1 mg/ml propidium iodide at room temperature in the dark for 30 min. The DNA content was then detected using a flow cytometer (BD Bioscience, Franklin Lakes, NJ, USA).

### 2.6. Apoptosis and Necrosis Assay

Ca9-22 and HSC-3 cells (1 × 10^3^ cells/well) were seeded in 96-well plates with DMEM containing 10% FBS for 24 h. The attached cells were treated with the various concentrations of HABE in serum-free media for 24 h. A cell death detection ELISA kit (Roche Diagnostics, Mannheim, Germany) was used to assess apoptotic and necrotic cell death according to the manufacturer's instruction. The absorbance was measured at 405 nm using a microplate reader.

### 2.7. Western Blot Analysis

Human oral cancer Ca9-22 (5 × 10^5^ cells) and HSC-3 (1 × 10^6^ cells) cells were seeded in 100 mm culture dishes with DMEM containing 10% FBS for 24 h at 37°C in a humidified atmosphere with 5% CO_2_. The attached cells were treated with the various concentrations of HABE in serum-free DMEM for 24 h. The cells were lysed with RIPA buffer (Cell Signaling Technology) containing 1 mM phenylmethylsulfonyl fluoride and protease inhibitor cocktail (Roche, Mannheim, Germany). The cell lysates were centrifuged at 22,000 ×g for 15 min at 4°C, and the supernatants were quantitated using BCA protein assay reagents (Pierce, Rockford, IL). Cell extracts were separated using 12% sodium dodecyl sulfate-polyacrylamide gel electrophoresis and transferred to a polyvinylidene difluoride membrane (Millipore, Danvers, MA). The membrane was blocked with 10% skim milk and incubated with primary antibodies against specific target proteins in 3% skim milk, which was followed by incubation with horseradish peroxidase-conjugated secondary antibodies for 1 h. The target proteins were detected using an ECL kit (Amersham, Piscataway, NJ) according to the manufacturer's protocol. Densitometric analysis was performed using Image J software.

### 2.8. Osteoclast Formation

BMMs (5 × 10^4^ cells/well) were seeded in a 96-well plate and incubated with *α*-MEM containing 10% FBS, M-CSF (30 ng/ml), sRANKL (100 ng/ml), and different HABE concentrations for 5 days. Tartrate-resistant acid phosphatase- (TRAP-) positive multinucleated osteoclasts with more than three nuclei were counted under light microscopy (×100 magnification) as previously described [15].

### 2.9. Statistical Analysis

The data are expressed as the means ± standard error (SE). One-way analysis of variance (ANOVA) was performed for statistical analysis, and Student's *t*-test was used to evaluate the differences between each group. *P* values less than 0.05 were considered statistically significant.

## 3. Results and Discussion

Oxidative stress induced by ROS is closely related to cancer development as a result of DNA damage and the subsequent mutations [16]. ROS enhances bone resorption as it serves as second messengers in osteoclastogenesis-related signaling pathways [17, 18]. Thus, natural products with antioxidant activity can be promising agents for controlling both cancer growth and the related bone destruction. On the other hand, several studies have demonstrated that polyphenols in the presence of metal ions can induce DNA degradation and consequently result in cell death by acting as prooxidants [19, 20].

**Figure 1:**
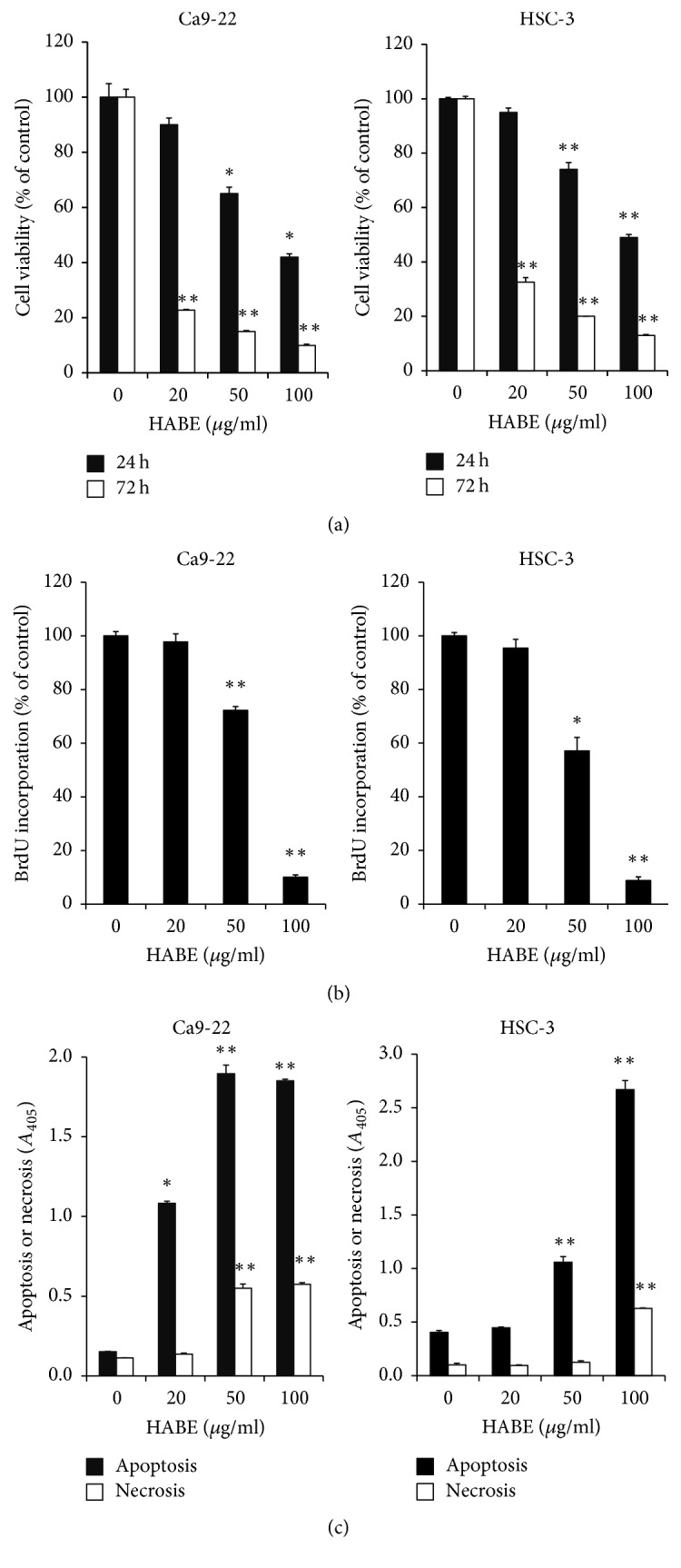
The inhibitory effect of HABE on the growth of oral cancer cells. Ca9-22 and HSC-3 cells (1 × 10^3^ cells/well) were cultured in serum-free media with the indicated HABE concentrations. (a) After 24 or 72 h of treatment with HABE, the cell viability was determined using an MTT assay. (b) After 24 h of treatment, the amount of newly synthesized DNA was evaluated using a BrdU incorporation assay, and (c) apoptosis and necrosis were assessed with a Cell Death Detection ELISA kit. Data are expressed as the mean ± SE. ^*∗*^*P* < 0.05 and ^*∗∗*^*P* < 0.001 versus vehicle-treated cells.

We first assessed the anticancer activity of HABE on OSCC by investigating its antiproliferative and apoptosis-inducing activity in two OSCC cell lines. Treatment with HABE for 24 h inhibited the viabilities of Ca9-22 gingival and HSC-3 tongue SCC cells by 35% and 26%, respectively, at 50 *μ*g/ml and 58% and 51%, respectively, at 100 *μ*g/ml (IC_50_ = 83 *μ*M for Ca9.22 and 98 *μ*M for HSC-3). The cell viabilities were inhibited by 77% and 67%, respectively, after 72 h of treatment at 20 *μ*g/ml (IC_50_ = 1.6 *μ*M for Ca9.22 and 1.7 *μ*M for HSC-3) (Figure 1(a)). Abnormally promoted cell proliferation and dysregulated apoptosis contribute to the unlimited proliferative potential of cancer [21]. We confirmed that HABE reduced the proliferation of OSCC cells by measuring the reduced BrdU incorporation in two HABE-treated OSCC cell lines. Treatment with 50 *μ*g/ml HABE for 24 h reduced the proliferation by 28% in Ca9-22 cells and 43% in HSC-3 cells. Treatment with 100 *μ*g/ml extract inhibited the proliferation of these cells by more than 90% in both OSCC cell lines (Figure 1(b)). Apoptotic and necrotic cell death were analyzed by measuring the histone-associated DNA fragments in cell lysates and culture media, respectively, from Ca9-22 and HSC-3 cells that were treated with HABE. Apoptotic cell death was increased 7.1- and 12.5-fold in Ca9-22 cells treated with 20 *μ*g/ml and 50 *μ*g/ml HABE, respectively, and 2.6- and 6.6-fold in HSC-3 cells treated with 50 *μ*g/ml and 100 *μ*g/ml HABE, respectively. Lower levels of necrotic cell death were observed compared to apoptotic cell death in Ca9-22 cells that were treated with more than 50 *μ*g/ml HABE and in HSC-3 cells that were treated with 100 *μ*g/ml HABE (Figure 1(c)). These results indicate that HABE treatment can inhibit the growth of OSCC cells primarily by blocking cell proliferation and inducing apoptosis in Ca9-22 and HSC-3 cells.

**Figure 2:**
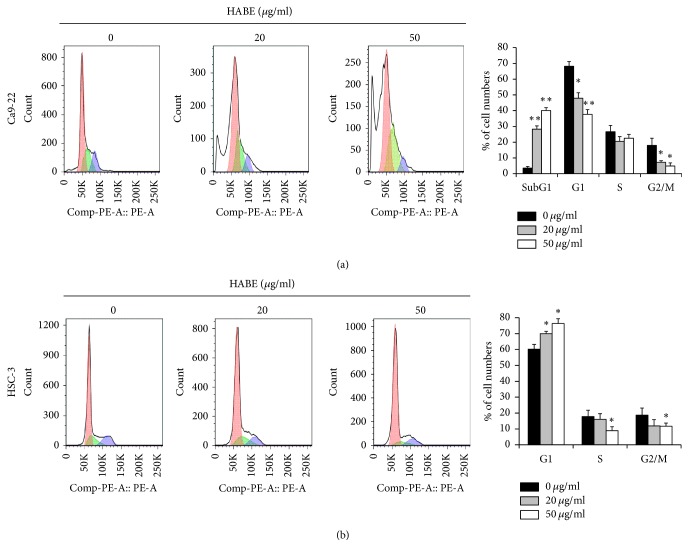
Cell cycle distribution in HABE-treated OSCC cells. (a) Ca9-22 or (b) HSC-3 cells were treated with different HABE concentrations in serum-free media for 24 h. The cells fixed with ice-cold 70% ethanol were incubated in propidium iodide solution for 30 min as described in the Materials and Methods section. DNA content was detected using a flow cytometer. Data are expressed as the mean ± SE. ^*∗*^*P* < 0.05 and ^*∗∗*^*P* < 0.001 versus vehicle-treated cells.

To further verify the growth inhibitory activity of HABE, we examined whether this extract could arrest the cell cycle in Ca9-22 and HSC-3 cells. Flow cytometric analysis showed that the sub-G1 peak was noticeably increased in HABE-treated Ca9-22 cells (Figure 2(a)) and cell populations at the G1 phase was dose-dependently enhanced in HABE-treated HSC-3 cells (Figure 2(b)). Therefore, treatment with this extract may strongly induce apoptotic cell death in Ca9-22 cells. In HSC-3 cells, HABE treatment may trigger apoptotic cell death via cell cycle arrest at the G1 phase.

**Figure 3:**
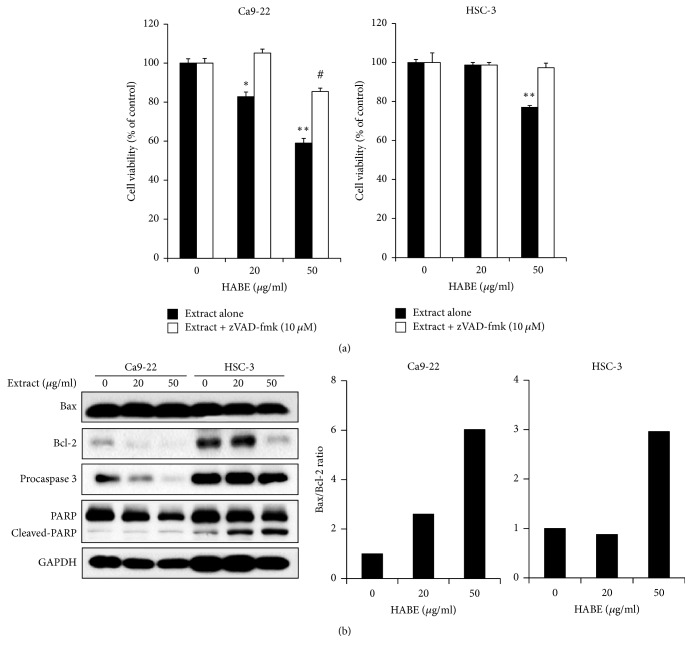
Induction of apoptotic cell death in HABE-treated oral cancer cells. (a) Ca9-22 and HSC-3 cells (1 × 10^3^ cells/well) were treated with the indicated HABE concentrations in the presence of a pan-caspase inhibitor, zVAD-fmk (10 *μ*M), for 24 h. Cell viability was assessed using an MTT assay. (b) Ca9-22 and HSC-3 cells were treated with the indicated HABE concentrations for 24 h. The levels of Bax, Bcl-2, procaspase 3, and full-length and cleaved PARP were detected by western blot analysis with specific antibodies.* The images are representatives of three independent experiments*. The ratio of Bax to Bcl-2 was determined after normalization with the GAPDH intensity by densitometry. Data are expressed as the mean ± SE. ^*∗*^*P* < 0.05 and ^*∗∗*^*P* < 0.001 versus vehicle alone-treated cells; ^#^*P* < 0.001 versus the cells treated with vehicle and zVAD-fmk.

We studied the molecular mechanism by which HABE could stimulate apoptotic cell death. The apoptosis signaling pathway is transduced through the intrinsic pathway (mitochondrial-dependent) or extrinsic (death receptor-dependent) pathway. In particular, the intrinsic pathway via the Bcl-2 family members, including the proapoptotic proteins (e.g., Bax, Bad, and Bak) and antiapoptotic proteins (e.g., Bcl-2 and Bcl-xL), play a pivotal role in transducing apoptotic signals [21, 22]. Among them, the ratio of proapoptotic Bax to antiapoptotic Bcl-2 can be a checkpoint for assessing apoptosis and is positively correlated with apoptosis induction [23]. Mitochondrial malfunction, which is expedited by pro- and antiapoptotic Bcl-2 family proteins, causes cytochrome *c* release. The released cytochrome *c* forms an apoptosome with caspase 9 and Apaf-1 and triggers the caspase cascade, including caspase 3 activation [24]. The activated caspase 3 cleaves poly(ADP-ribose) polymerase (PARP), which is associated with DNA damage repair [21, 25, 26]. In our study, HABE treatment in the presence of zVAD-fmk, a pan-caspase inhibitor, rescued the cell viabilities in both OSCC cell lines, suggesting that caspase activation is required for HABE-induced apoptosis (Figure 3(a)). In turn, we confirmed that treatment with HABE elevated the Bax to Bcl-2 ratio by reducing Bcl-2 protein expression and resulted in the caspase 3 activation and PARP cleavage in Ca9-22 and HSC-3 cells (Figure 3(b)). These results indicate that HABE extract induces apoptosis through the intrinsic pathway in Ca9-22 and HSC-3 cells. Low Bcl-2 level may affect the responsiveness of Ca9.22 cells on HABE treatment.

**Figure 4:**
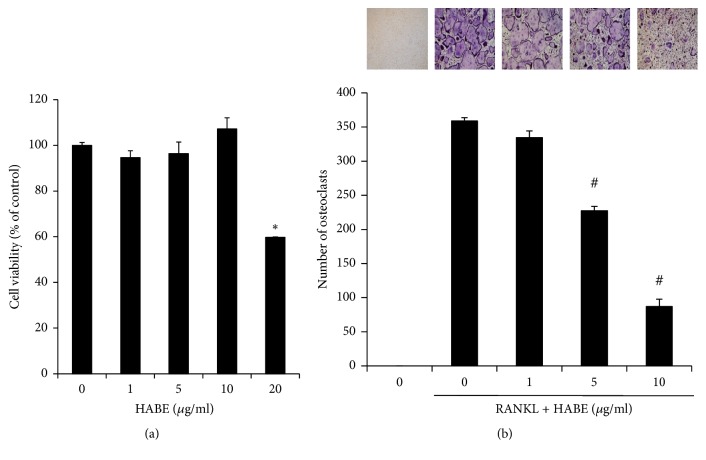
The inhibitory effect of HABE on RANKL-induced osteoclastogenesis. (a) BMMs (5 × 10^4^ cells/well) were treated with the indicated HABE concentrations for 5 days in *α*-MEM containing 10% FBS and M-CSF (30 ng/ml). The cell viability was determined using an MTT assay. (b) BMMs (5 × 10^4^ cells/well) were incubated with HABE at the indicated concentrations in the presence of 10% FBS, M-CSF (30 ng/ml), and RANKL (100 ng/ml) for 5 days. TRAP-positive multinucleated osteoclasts with more than three nuclei were counted. Data are expressed as the mean ± SE. ^*∗*^*P* < 0.001 versus BMMs treated with M-CSF alone. ^#^*P* < 0.001 versus BMMs treated with M-CSF and RANKL.

Bone resorption occurring in the maxillary or mandibular bone of OSCC patients is mediated by osteoclasts rather than by the carcinoma itself. OSCC cell-derived factors promote the production of RANKL in osteoblastic/stromal cells, and this RANKL promotes the formation of mature osteoclasts from osteoclast precursors. Bone resorption by mature osteoclasts releases growth factors from mineralized matrix and consequently multiplies tumor growth and the production of osteolytic factors [27]. We examined the antiosteoclastogenic activity of HABE in RANKL-stimulated BMMs. Treatment with HABE inhibited the viability of BMMs by 40% at 20 *μ*g/ml (Figure 4(a)) and inhibited RANKL-induced osteoclast formation by 37% at 5 *μ*g/ml and 76% at 10 *μ*g/ml (IC_50_ = 6.1 *μ*M) (Figure 4(b)).

## 4. Conclusion

HABE inhibited the proliferation by arresting cell cycle at the G1 phase and promoted apoptosis via intrinsic (mitochondrial) pathway in OSCC cells. In addition, HABE blocked RANKL-induced osteoclastogenesis at noncytotoxic concentrations. A clinical pharmacology study reported that 70% of clinical symptoms were improved in the individuals given 4 g of* H. antidysenterica* stem bark extract/day [11]. Therefore, HABE may be a safe anticancer agent against OSCC growth and OSCC-mediated bone resorption. Further studies are required to identify major active components of HABE and to determine their in vivo activities.
